# Neuroendocrine Inflammatory Responses in Overweight/Obese Infants

**DOI:** 10.1371/journal.pone.0167593

**Published:** 2016-12-01

**Authors:** Ana Cristina Resende Camargos, Vanessa Amaral Mendonça, Camila Alves de Andrade, Katherine Simone Caires Oliveira, Rosalina Tossige-Gomes, Etel Rocha-Vieira, Camila Danielle Cunha Neves, Érica Leandro Marciano Vieira, Hércules Ribeiro Leite, Murilo Xavier Oliveira, Antônio Lúcio Teixeira Júnior, Cândido Celso Coimbra, Ana Cristina Rodrigues Lacerda

**Affiliations:** 1 Departamento de Fisioterapia, Universidade Federal dos Vales do Jequitinhonha e Mucuri, Diamantina, Minas Gerais, Brazil; 2 Programa Multicêntrico de Pós-Graduação em Ciências Fisiológicas, Sociedade Brasileira de Fisiologia, Diamantina, Brazil; 3 Faculdade de Medicina, Universidade Federal de Minas Gerais, Belo Horizonte, Minas Gerais, Brazil; 4 Instituto de Ciências Biológicas, Universidade Federal de Minas Gerais, Belo Horizonte, Minas Gerais, Brazil; East Tennessee State University, UNITED STATES

## Abstract

Childhood obesity is related to a cascade of neuroendocrine inflammatory changes. However, there remains a gap in the current literature regarding the possible occurrence of these changes in overweight/obese infants. The objective of this study was to evaluate adipokines, cortisol, brain-derived neurotrophic factor (BDNF) and redox status in overweight/obese infants versus normal-weight peers. A cross-sectional study was conducted with 50 infants (25 in the overweight/obese group and 25 in the normal-weight group) between 6 and 24 months. Plasma levels of leptin, adiponectin, resistin, soluble tumor necrosis factor (TNF) receptors, chemokines, BDNF, serum cortisol and redox status were measured. Unpaired Student's t-test was used to analyze the results and a probability of p<0.05 was acceptable for rejection of the null hypothesis. The Pearson correlation was used to verify the association between the biomarkers analyzed in each group. Plasma levels of leptin (p = 0.0001), adiponectin (p = 0.0007) and BDNF (p = 0.003), and serum cortisol (p = 0.048) were significantly higher in overweight/obese infants than normal-weight infants. In contrast, the concentration of thiobarbituric acid reactive substances (TBARS) (p = 0.004), and catalase (p = 0.045) and superoxide dismutase activity (p = 0.02) were lower in overweight/obese infants than normal-weight peers. All the results together indicate neuroendocrine inflammatory response changes in overweight/obese infants between 6 and 24 months. Although there is already an environment that predisposes for a subsequent pro-inflammatory response, neuroendocrine secretion changes that permit the control of the inflammatory process in this age interval can be observed.

## Introduction

Obesity is defined as a long-term positive caloric imbalance leading to adipose tissue expansion, which may occur via increases in adipocyte size (hypertrophy), number (hyperplasia) or both [1,2]. Obese individuals presented a chronic low-grade inflammation that involves excessive adipocyte hypertrophy, immune cell infiltration, extracellular matrix overproduction, increased production of pro-inflammatory adipokines and redox imbalance [3,4,5].

The obesity and overweight dysfunctional adipose tissue releases chemokines that attract circulating monocytes and other leukocyte subsets to infiltrate adipose tissue, contributing to the altered adipokine secretion pattern [3,4,6]. As a result, pro-inflammatory cytokines, such as the tumor necrosis factor (TNF), considered to be a major macrophage-derived paracrine mediator of inflammation in the adipose tissue, are overproduced [3,4]. Higher levels of soluble TNF receptors, resistin and leptin, as well as lower levels of adiponectin are related to inflammation, insulin resistance and cardiovascular diseases [7,8].

Hypertrofic adipose tissue growth is associated with adipose tissue inflammation [2]. Therefore, an increase in pro-inflammatory adipokines secreted by infiltrated macrophages and hypertrophied adipocytes, as well as an elevated production of reactive oxygen species (ROS), can decrease the differentiation capacity of preadipocytes and inhibit adipogenesis [4,5,9,10]. On the other hand, the current literature has demonstrated that hyperplastic growth, generating healthy, functional, smaller adipocytes, maintains the functioning of healthy adipose tissue through enhanced recruitment of adipocyte precursor cells that are differentiated into small adipocytes, with minimal inflammation and redox imbalance [1,9]. Thus, higher levels of adiponectin, an anti-inflammatory adipokine, can inhibit pro-inflammatory effects and can help to maintain equilibrium adipocyte, size-accelerating adipogenesis [5,11,12]. Moreover, other endocrine and autocrine factors can mediate the equilibrium adipocyte size, interfering in the synthesis and activity of adipogenic transcription factors as hormones and growth factors. Among these, cortisol is considered to be an important hormone inducer of adipogenesis [13], and brain-derived neurotrophic factor (BDNF) plays a regulatory role in human adipocyte differentiation [14]. Childhood obesity is a serious problem of public health, and the prevalence is increasing in very young children, including infants [15]. Early postnatal weight gain in infancy is associated with overweight and obesity, central adiposity, low-grade systemic inflammation and redox imbalance in later childhood, predisposing to metabolic derangements and chronic inflammatory diseases like insulin resistance and cardiovascular impairment [16,17,18].

Several studies have demonstrated the dysregulated secretion pattern of these biomarkers in childhood. Higher levels of leptin, resistin, soluble TNF receptors 1 (sTNFR1) and 2 (sTNFR2), monocyte chemoattractant protein-1 (MCP-1), regulated upon activation normal T-cell expressed and secreted (RANTES), interleukin-8 (IL-8), interferon-inducible protein 10 (IP-10), monokine induced by interferon-γ (MIG), ROS and cortisol, as well as lower levels of adiponectin, BDNF and antioxidant enzymes in school-age overweight or obese children can be observed [8,10,19,20,21,22,23,24,25]. Another study already demonstrated elevated C-reactive protein (CRP) levels, another inflammatory marker, in preschool obese children [26]. Thus, obese children over two years of age present excessive adipocyte hypertrophy and hyperplasia compared to normal-weight children [17,27]. The larger sized adipocytes in childhood obesity already seem able to trigger a cascade of neuroendocrine inflammatory changes to activate a chronic low-grade inflammation state and redox imbalance.

However, no data about inflammatory, hormonal and neurotrophic markers in infants up to the age of 2 years exists. There is a gap in the current literature regarding the possible occurrence of these changes in overweight and obese infants. Considering that infancy is a period of accelerated accumulation of adipose tissue mass [17,27], it is important to verify whether the expansion of adipose tissue promotes all these changes in this age group.

The objective of this study was to evaluate the plasmatic levels of adipokines [leptin, adiponectin, resistin, sTNFR1, sTNFR2 and chemokines (MCP-1, RANTES, IL-8, IP-10 and MIG)] and BDNF, serum cortisol and redox status in overweight/obese infants versus normal-weight peers matched for gender, age, socioeconomic status, maternal education, exclusive breastfeeding until 6 months, and the use of vitamin supplements. It is believed that there can already be changes in the secretion of neuroendocrine inflammatory parameters in overweight/obese children in this early stage of infant development. These changes: 1). contribute to the low-grade inflammation and oxidative stress in childhood; or 2). promote balance towards controlling or inhibiting the low-grade inflammatory status and oxidative stress in infancy.

## Materials and Methods

### Study design and subjects

A cross-sectional study was conducted with infants between 6 and 24 months enrolled in Family Health Strategies in the city of Diamantina, Minas Gerais, Brazil. The overweight/obese group included infants with body mass index (BMI) ≥ 97th percentile (z score > +2). For each overweight/obese infant, a normal-weight peer (BMI ≥ 3 and < 85th percentile: z score > -2 and < +1) was selected to match for gender, age, socioeconomic status, maternal education, exclusive breastfeeding until 6 months and use of vitamin supplements. These participants were classified on the basis of World Health Organization (WHO) BMI-for-age cut-off points relative to age and gender [28]. Exclusion criteria were as follows: preterm infants; infants with signs of malnutrition or illness that interfere with growth and development; infants who had some infectious process (fever, influenza, diarrhea, ear infections, etc.) or who were vaccinated in the past 15 days, and infants using any medication. This study was approved by the Scientific Ethics Committee of the Federal University of Jequitinhonha and Mucuri Valleys (protocol n^o^. 085/12). A visit to the home of the selected infants was performed to explain to parents and/or guardians the study’s objectives and procedures. Those parents who agreed to participate signed a consent form.

### Assessment of BMI and infant data

BMI was calculated on the basis of body weight and body length measurements. A pediatric electronic scale (Welmy, São Paulo, Brazil) with a 15-kg capacity and an accuracy of 5 g was used to weigh the infants. A portable infant stadiometer (Seca, Hamburg, Germany) with millimeter resolution was used to measure infant length. These measurements were performed by the same, properly trained examiner. The WHO Anthro software version 3.2.2 (Geneva, Switzerland) was used to calculate BMI-for-age, expressed as z-scores [29].

The parents answered questions about exclusive breastfeeding time, use of vitamin supplement and infant health data. The Brazilian Criteria of Economic Classification, developed by the Brazilian Association of Research Companies, was used to assess the socioeconomic status of families. Classes were grouped into A, B, C, D and E, in which A indicates the highest economic class and E the lowest economic class [30].

### Measurements of plasma adipokines, BDNF and serum cortisol

Twenty-four hours after the initial assessment, six milliliters of each of the blood samples was collected in a local laboratory following 3 hours of fasting in the morning. The tubes (sodium heparin and serum) were centrifuged to remove cells and debris and were stored as plasma, serum and erythrocytes aliquots at -80°C until use.

Plasma leptin, adiponectin, resistin, BDNF and soluble TNF receptor (sTNFR1, sTNFR2) levels were measured using conventional sandwich ELISA kits (DuoSet, R&D Systems, Minneapolis, MN, USA), according to the manufacturer's instructions. The detection limits were 5.0 pg/mL for all the kits.

Plasma chemokines (CCL2/MCP-1, CCL5/RANTES, CXCL8/IL-8, CXCL10/IP-10 and CXCL9/MIG) were measured using the cytometric bead arrays kit (BD Bioscience, San Jose, CA, USA) according to the manufacturer’s protocol. Bead flow cytometry allows the simultaneous quantification of various proteins in the same test. Samples were acquired in a FACSCanto flow cytometer (BD Biosciences, San Jose, CA, USA) and analyzed using the FCAP Array v1.0.1 software (Soft Flow Inc.). The detection limits were 0.2 pg/mL for IL-8, 1.0 pg/mL for RANTES, 0.2 pg/mL for MIG, 2.7 pg/mL for MCP-1 and 2.8 pg/mL for IP-10.

Serum cortisol was measured using competitive ELISA kit (IBL, AMERICA, Minneapolis, MN, USA) according to manufacturer’s instructions. The detection limits were 55.2 nmol/L.

### Measurements of redox status

The redox status was assessed by thiobarbituric acid reactive substances (TBARS) concentration, antioxidants enzymes dismutase superoxide (SOD) and catalase (CAT) activity, as well by ferric reducing antioxidant power (FRAP) in the erythrocyte lysate. The erythrocyte lysate was prepared as described by Glass and Gershon [31]. Protein concentration of samples was determined by the Bradford method [32] using bovine serum albumin (BSA) (1 mg/mL) as standard.

TBARS concentration was measured according to the method described by Ohkawa et al. [33], and the reaction of the thiobarbituric acid with malondialdehyde (MDA) was used to determine lipid peroxidation. TBARS concentration, expressed in nmol MDA/mg protein, was determined from the standard curve constructed with known concentrations of MDA (1,1,3,3-tetramethoxypropane) (Sigma, USA). The assay to determine SOD (EC 1.15.1.1) activity was performed according to Srivastava et al. [34] and expressed in U/mg of protein. SOD activity was determined by measuring the capacity of SOD to inhibit the autoxidation of pyrogallol. CAT (EC 1.11.1.6) activity was measured according to the method of Nelson and Kiesov [35] and expressed by ΔE/min/mg protein, where ΔE represents the variation in enzyme activity during 1 minute. The total antioxidant capacity (FRAP) was determined according to the method of Benzie and Strain [36], which is based on the reduction of ferric-tripyridyltriazine [Fe(III)-TPTZ] complex to ferrous-tripyridyltriazine [Fe(II)-TPTZ]. The total antioxidant capacity was expressed as equivalents of Fe^2+^, estimated by comparison with a standard curve constructed with known concentrations of FeSO_4_ and expressed as μg FeSO_4_/mg of protein.

### Statistical analyses

The data were analyzed using the SPSS statistical package, version 17.0 (Inc., USA) and GraphPad Prism, version 5.0 (Inc., USA). The Chi-square test or Fisher's exact test were utilized to compare the proportions of the two groups. The Shapiro-Wilk and Levene test were applied to evaluate the normality and homogeneity of results, respectively. Log-transformations were used to normalize the BDNF, MCP-1, RANTES, IL-8, IP-10, MIG, sTNFR1, sTNFR2, TBARS, CAT, SOD and FRAP data. The Student’s unpaired t-test was performed to compare the groups, and the Pearson correlation was used to verify the association between the biomarkers analyzed in each group. The significance level was 5% (α< 0.05).

A sample size of 50 participants (25 in the overweight/obese group and 25 in the normal-weight group) was required to test a minimum 0.34 effect size with a power equal to 90% and a two-tailed α-value = 0.05 for the leptin, adiponectin, BDNF, sTNFR1, sTNFR2, MCP-1, RANTES, IL-8, IP-10 and MIG variables. At least 20 participants in each group was required for the test with cortisol, and at least 18 participants in each group was required for the TBARS, CAT, SOD and FRAP variables to obtain a power equal to 90% and a two-tailed α-value = 0.05.

## Results

### Characteristics of study participants

The groups were similar in gender, economic status, maternal education, exclusive breastfeeding until 6 months, use of vitamin supplements, age and body length. Body weight and BMI values were significantly higher in the overweight/obese group (Table 1) than in the normal-weight group.

**Table 1:** Sample characterization.

Sample characterization			Normal-weight(n = 25)	Overweight/obese(n = 25)	p
Gender		F(%)			1.00^a^
	Boys		16 (50.0%)	16 (50.0%)	
	Girls		9 (50.0%)	9 (50.0%)	
Socioeconomic status		F(%)			1.00^b^
	B		4 (57.1%)	3 (42.9%)	
	C		17 (50.0%)	17 (50.0%)	
	D		4 (44.4%)	5 (55.6%)	
Maternal education		F(%)			0.76^b^
	Incomplete primary school		4 (57.1%)	3 (42.9%)	
	Incomplete high school		7 (51.3%)	5 (41.7%)	
	Incomplete higher education		13 (48.1%)	14 (51.9%)	
	Higher education diploma		1 (25.0%)	3 (75.05)	
Exclusive breastfeeding until 6 months		F(%)			0.57^a^
	Yes		10 (43.5%)	13 (56.5%)	
	No		15 (55.6%)	12 (44.4%)	
Vitamin supplement		F(%)			1.00^b^
	Yes		4 (50.0%)	4 (50.0%)	
	No		21 (50.0%)	21 (50.0%)	
Age (days)					0.99^c^
	Mean (±SEM)		357.88 (±30.07)	358.00 (±29.91)	
Body weight (Kg)					0.0001^c^*
	Mean (±SEM)		8.97 (±0.32)	12.19 (±0.55)	
Body length (cm)					0.28^c^
	Mean (±SEM)		73.30 (±1.43)	75.55 (±1.52)	
BMI (Kg/m^2^)					
	Mean (±SEM)		16.62 (±0.21)	21.04 (±0.23)	0.0001^c^*

F, frequency; SEM, standard error mean; BMI, body mass index.

^a^Chi-Square

^b^Fisher’s exact test

^c^Student’s unpaired t-test.

*Significant difference (p<0.05).

### Plasma and serum biomarkers levels

**Fig 1:**
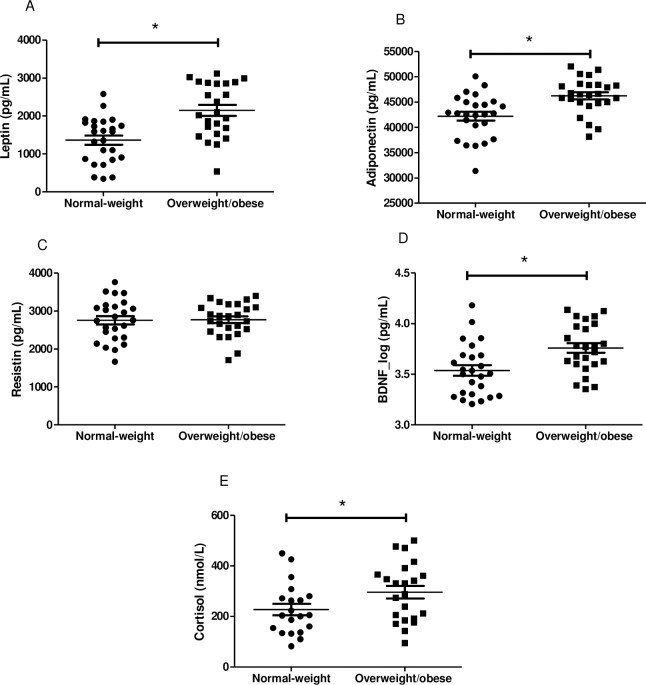
Plasma levels of leptin, adiponectin, resistin, BDNF and serum level of cortisol in two groups. A-D: n = 25 in each group. D: BDNF was expressed in log. E: n = 22 in overweight/obese and n = 20 in normal-weight group. Values are expressed as mean±SEM. *Differences were considered to be significant at p<0.05.

Plasma levels of leptin (p = 0.0001; 95% CI: -1164, -404.6), adiponectin (p = 0.0007; 95% CI: -6289, -1810) and log BDNF (p = 0.003; 95% CI: -0.37, -0.08) were significantly higher in overweight/obese infants than in normal-weight peers. Serum cortisol also presented higher levels in overweight/obese infants (p = 0.048; 95% CI: -136.4, -0.47). However, there was no significant difference between the two groups in mean values of plasma resistin (p = 0.90; 95% CI: -299.4, 263.3) (Fig 1).

**Fig 2:**
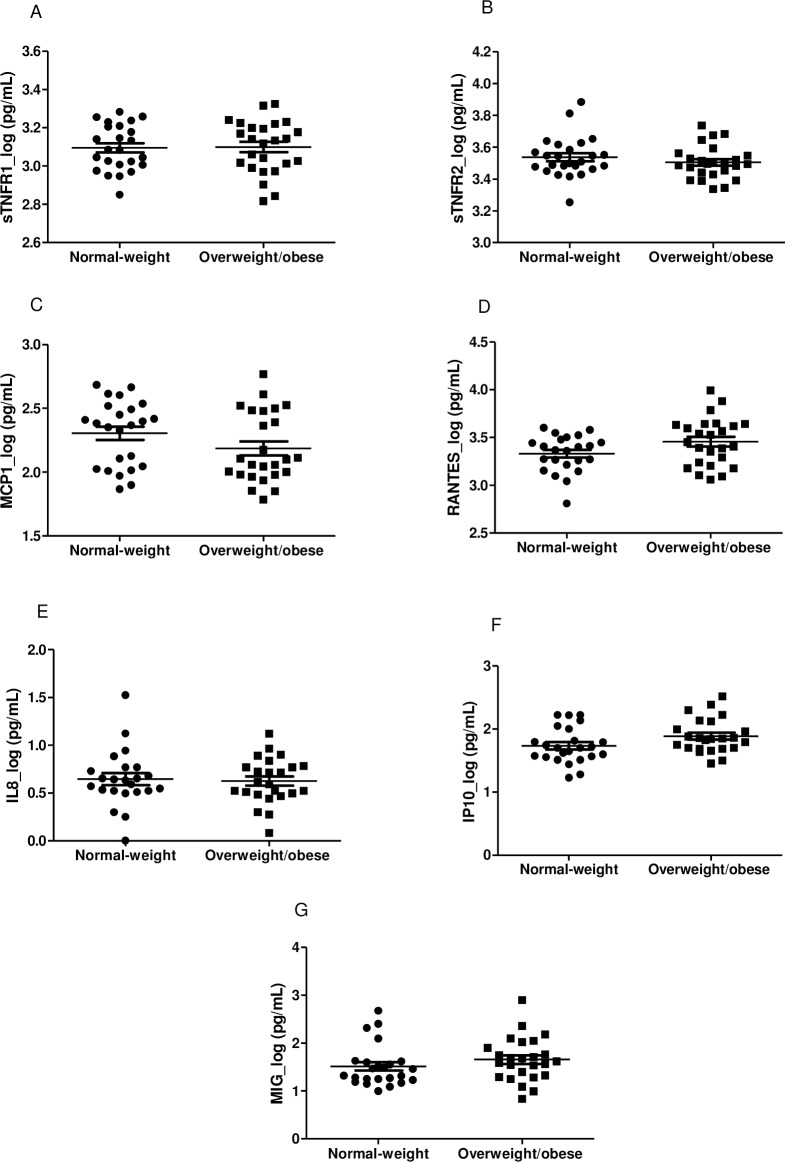
Plasma levels of soluble receptors of TNF (sTNFR1 and sTNFR2) and chemokines (MCP-1, RANTES, IL-8, IP-10 and MIG) in normal-weight and overweight/obese infants. A-G: n = 25 in each group. Values are expressed in log as mean±SEM.

Soluble receptors of TNF (sTNFR1 (p = 0.92; 95% CI: -0.08, 0.07) and sTNFR2 (p = 0.32; 95% CI: -0.03, 0.09)) and chemokines [MCP-1 (p = 0.13; 95% CI: -0.04, 0.27), RANTES (p = 0.06; 95% CI: -0.26, 0.01), IL-8 (p = 0.79; 95% CI: -0.14, 0.18), IP-10 (p = 0.06; 95% CI: -0.31, 0.01) and MIG (p = 0.27; 95% CI: -0.40, 0.12)] log plasma levels did not differ significantly between the groups (Fig 2).

### Redox status

**Fig 3:**
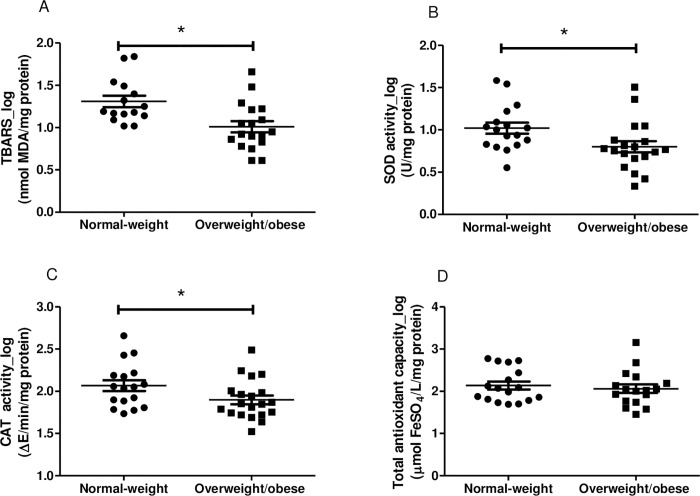
Redox status in normal-weight and overweight/obese infants. A-D: n = 18 in each group. Values are expressed in log as mean±SEM. *Differences were considered to be significant at p <0.05.

With regard to redox status, significant differences were found in TBARS concentrations (p = 0.004; 95% CI: 0.10, 0.49) and CAT (p = 0.045; 95% CI: 0.003, 0.34) and SOD (p = 0.02; 95% CI: 0.03, 0.41) activity. The levels for the overweight/obese group were lower than those of their normal-weight peers. No significant difference in mean values of FRAP (p = 0.59; 95% CI: -0.21, 0.36) (Fig 3) was found between the two groups.

### Correlation between biomarkers analyzed

In overweight/obese infants, the adiponectin plasma levels were inversely associated with leptin (p = 0.02; r = -0.61), sTNFR1 (p = 0.03; r = -0.45) and IL8 (p = 0.008; r = -0.53). There was also a negative association of cortisol with sTNFR1 (p = 0.04; r = -0.44) and MCP-1 (p = 0.02; r = -0.49). BDNF was positively associated with sTNFR1 (p = 0.03; r = 0.43), MCP-1 (p = 0.03; r = 0.44), IL-8 (p = 0.009; r = 0.52) and IP-10 (p = 0.04; r-0.42). Positive associations between multiple biomarkers with pro-inflammatory roles were also demonstrated: leptin and sTNFR1 (p = 0.02; r = 0.46); resistin and sTNFR2 (p = 0.03; r = 0.43); sTNFR1 and sTNFR2 (p = 0.007; r = 0.52); sTNFR2 and IP-10 (p = 0.02; r = 0.46); IL8 and RANTES (p = 0.04; r = 0.41); MIG and RANTES (p = 0.01; r = 0.50); MIG and IL-8 (p = 0.007; r = 0.54) and; IP-10 and MIG (p = 0.0001; r = 0.83). Finally, TBARS were positively associated with SOD (p = 0.04; r = 0.52) and CAT (p = 0.03; r = 0.54) activity; and the antioxidant defense was also positively associated [CAT and SOD (p = 0.0001; r = 0.84); SOD and FRAP (p = 0.0001; r = 0.83); CAT and FRAP (p = 0.0001; r = 0.82)].

Positive associations between plasma levels of pro-inflammatory biomarkers were observed in the normal weight group: resistin and IP-10 (p = 0.02; r = 0.59); resistin and MIG (p = 0.03; r = 0.46); sTNFR1 and sTNFR2 (p = 0.0001; r = 0.67); sTNFR1 and IP-10 (p = 0.01; r = 0.51); sTNFR1 and MIG (p = 0.02; r = 0.47); sTNFR2 and MIG (p = 0.007; r = 0.53); IL-8 and MIG (p = 0.002; r = 0.60); IP-10 and MIG (p = 0.02; r = 0.48). A positive association of TBARS with the activity of SOD (p = 0.0001; r = 0.85) and CAT (p = 0.02; r = 0.67) was demonstrated. The activities of SOD and CAT enzymes also had a positive association with each other (p = 0.04; r = 0.53).CAT was also had positive associated with FRAP (p = 0.03; r = 0.58). Finally, A positive association of FRAP with BDNF (p = 0.03; r = 0.56) was observed

## Discussion

This study showed that overweight or obese infants between 6 and 24 months presented higher levels of leptin, adiponectin, BDNF, cortisol and lower levels of TBARS, CAT and SOD activity than those of their normal-weight peers. There were no differences in the levels of resistin, soluble TNF-α receptors, chemokines and FRAP between the groups. As far as it is known, this is the first study that evaluated all these biomarkers in infancy. These results indicate neuroendocrine inflammatory response changes in overweight/obese infants. Moreover, it is possible to show that neuroendocrine secretion changes occur to control the inflammatory process in this age interval.

The increase in plasma leptin levels found in this study was expected in overweight/obese infants that presented a higher body weight and BMI than normal-weight peers. Leptin is an adipokine that acts by signaling body fat stores to the hypothalamus, which, in turn, regulates food intake and energy expenditure to maintain body weight homeostasis [37]. Higher circulating concentrations of leptin may inhibit enlargement of fat depot size and influence the number of cells present through the inhibition of preadipocyte proliferation or reduction in adipocyte size [38]. However, elevated levels of leptin may induce monocyte activation, cytokine production and redox imbalance [5]. In the present study, the leptin levels were positively associated with sTNFR1 levels and negatively associated with adiponectin levels in overweight/obese infants, showing that the increase in plasma leptin levels can create an environment that predisposes to a subsequent pro-inflammatory response.

The plasma levels of adiponectin were also high in the overweight/obese group. This result is different from that of other studies that found lower circulating adiponectin levels in school-aged obese children [20,39]. However, adiponectin is not only related to the amount of body fat, but also the maturational stage of the adipose tissue [40]. Adiponectin levels are high at birth, they are positively correlated with adiposity in neonatal period [41], and they decreased longitudinally with increasing age and adiposity in childhood and adulthood [42]. These elevated levels of adiponectin in infancy can help to protect overweight/obese infants against several diseases, because adiponectin has anti-inflammatory and anti-atherogenic effects and augments insulin sensitivity, thereby protecting against obesity-related cardiovascular and metabolic diseases [11,12,43]. In the present study, plasma adiponectin levels were inversely associated with the plasma pro-inflammatory biomarkers—leptin, sTNFR1 and IL8 levels—in the overweight/obese group. This fact reinforces the suggestion that adiponectin exerts a possible anti-inflammatory protective effect.

In addition to adiponectin, cortisol was also present in high concentrations in overweight/obese infants. Cortisol can tune inflammatory responses by suppressing immune or inflammatory genes [6,13]. Serum cortisol levels also showed a negative association with sTNFR1 and MCP-1 levels in the overweight/obese group. Cortisol could reduce chemokine expression, macrophage recruitment and decrease adipose tissue macrophage accumulation [44]. In addition, adiponectin functions as a direct regulator of the macrophage phenotype and favors the switch from a pro-inflammatory state (M1) to an anti-inflammatory state (M2) that secretes anti-inflammatory cytokines while it down-regulates the production of pro-inflammatory cytokines, chemokines and ROS [5,6,12,43].

Thus, higher plasma adiponectin and serum cortisol levels may have impeded the increase in plasma pro-inflammatory markers like sTNFR1, MCP-1 and IL-8 in the overweight/obese infants. sTNFR1 is a dominant mediator of the TNF function [45] and the principal paracrine mediator of inflammation in the adipose tissue [3,4]. MCP-1 is considered to be pivotal for recruitment of circulating monocytes into adipose tissue that is switching to the pro-inflammatory state M1 macrophage [44] and IL-8 are involved in the recruitment of monocytes and other immune cells into adipose tissue [4,12]. In this study, no differences were found between the groups with respect to these plasma level biomarkers and resistin, sTNFR2, RANTES, IP-10 and MIG. Despite the fact that the current literature highlights the higher pro-inflammatory biomarkers in school-aged obese children [17,19,23,46], it appears that the low-grade inflammatory state has not yet been triggered in overweight/obese infants. Although evidence of increased levels of leptin, a pro-inflammatory adipokine, in overweight/obese infants has been observed, it is noteworthy that these findings seem to indicate that elevated levels of adiponectin and cortisol could contribute to the control of the low-grade inflammatory state.

Overweight/obese infants still exhibit higher concentrations of BDNF. Together with leptin, BDNF is also involved in regulatory hypothalamic pathways essential for control of body weight and energy homeostasis [47,48]. However, the levels of these biomarkers were not associated in the present study. In this study, plasma BDNF levels were positively associated with the plasma levels of sTNFR1, MCP-1, IL-8 and IP-10. BDNF is present at high levels in infancy and is expressed throughout the developing and mature central nervous system and in many peripheral tissues, including adipose tissue [49]. BDNF is thought to have a crucial role in modulating neuroinflammation by promoting neuroprotection because high levels can increase neuronal resistance to metabolic stress [47].

Overweight/obese infants presented lower TBARS levels and lower activities of antioxidant enzymes SOD and CAT. Studies show that adipocyte dysfunction resulting from excess body fat increases the production of ROS, which can damage cell membranes by a process called lipid peroxidation. This reaction leads to the formation of MDA, as is indicated by the elevated levels of TBARS in overweight or obese children [16,50]. However, the result of the biomarker of lipid peroxidation (TBARS) in this study was distinct from the evidence presented in the literature for school-aged children [16,22], and the reason for this difference is not completely understood. The lower SOD and CAT activities was similar to those of other studies, which demonstrated the presence of a low antioxidant defense in overweight and obese children [16]. The lower concentration of TBARS may have reduced the activities of antioxidant enzymes SOD and CAT because their production is stimulus-dependent. This association was demonstrated in the present study in both groups. In addition, the total antioxidant capacity, composed of endogenous and exogenous antioxidants, was not different between the groups. The overweight/obese infants seem to have a lower degree of lipid peroxidation and antioxidant defense than normal-weight infants. This fact instigates new studies to verify the possible causes of these findings.

Considering the biology and development of adipose tissue, the large secretion pattern of adiponectin and cortisol is related to the differentiation and proliferation of adipocytes [13,18,51]. In addition, BDNF also has a potential role as a regulator of human adipocyte differentiation [14]. Therefore, the high levels of adiponectin, cortisol and BDNF found in overweight/obese infants of this study suggest that the expansion of adipose tissue is associated with a large increase in the number of adipocytes at this age interval. Adipose tissue rapidly expands in infancy as a result of increased size and number of adipocytes, and it is mainly composed of small, newly differentiated adipocytes [51]. According to Laudes [52], first excess energy is stored as additional triglycerides in existing adipocytes and results in an enlargement of these cells, which is called hypertrophy. Secondly, if the number of fat cells is not sufficient to store increasing amounts of triglycerides, new adipocytes are generated by adipogenesis in a process described as hyperplasia. A classical study showed that in normal-weight infants between 6 and 12 months or age, fat depots primarily increase in cell size, with only minor contributions by the number of cells. After 12 months, a decrease in cell size was observed in normal-weight infants, and they displayed small increments in cell number until the age of 24 months, thereby demonstrating that this period is important for hypercellularity of adipose tissue [27]. However, no data were found regarding the expansion of adipose tissue in overweight or obese children until the age of 24 months, and a gap in the literature regarding this question remained. Although this study did not evaluate samples of adipose tissue that could confirm a possible expansion of adipose tissue in overweight/obese infants, these results lead us to believe that this expansion seems to have occurred through a balance of hypertrophy and hyperplasia, and a marked hypercellularity of adipose tissue.

The early hypercellularity in obese children is considered to be the strongest predictor of adipose tissue mass in childhood [17]. This fact recalls the earlier concept of a “hyperplasic” model of obesity in children versus a “hypertrophic” model in adults [53]. In the “hyperplasic” model, healthy adipose expansion with differentiation into small adipocytes and the presence of M2-like macrophages that help to maintain tissue homeostasis and preserve cellular functionality occurs [6,9,54]. In contrast, in the “hypertrophic” model, pathological adipose expansion consists of a massive enlargement of existing adipocytes, and M1-like macrophages prevail, leading to an inflammatory phenotype [54].

Finally, the dynamic secretions of adipokines, cortisol, and BDNF seem to be having different functions throughout growth and development during infancy, childhood and adulthood [40,53]. Therefore, it is important to note that, in spite of the fact that the early increase in levels of cortisol and adiponectin may partially protect against of low-grade inflammatory state in infancy, the long-term effect can predispose to other metabolic complications. Moreover, long-term elevated levels of leptin promote leptin resistance, leading to energy balance dysregulation and inducing a redox imbalance [5]. Chronic excess of cortisol produces increased body fat and promotes redistribution of human adipocytes from peripheral to central depots and an accumulation of visceral fat and abdominal obesity [13,18,44], that are related to cardiovascular risk factors, insulin resistance and development of the metabolic syndrome in obese children and adolescents [18,24]. Chronic elevated cortisol levels and leptin resistance can still explain the lower secretion of BDNF found in morbidly obese children and children with metabolic syndrome [25,48], and they are associated with deficits in cognition and synaptic plasticity [55]. Considering that adiponectin decreases longitudinally in childhood [39] and low adiponectin levels are predictive of the accumulation of cardiovascular risk factors and metabolic syndrome in obese school children [20], there is a long-term reduction in its protective role. Thus, it is possible that early modifications may contribute to the chronic low-grade inflammation and redox imbalance in childhood or adulthood.

It is still important to consider that some factors that could have affected the results of this study were controlled by matching between groups. Breastfeeding should be considered because several adipokines, cortisol and antioxidants have been identified in human breast milk [56,57]. The use of vitamin supplements for some infants, prescribed by pediatricians, also has influenced the results of the total antioxidant capacity and the level of oxygen free radicals [50].Furthermore, economic status could affect cortisol levels [58].

This study also had some limitations. First, BMI was used to classify the groups, and a more comprehensive examination of body composition would have been of great value to this study and should be considered in future studies in infants. Secondly, it was not possible to control the dietary patterns and certain nutritional factors that can modify the secretion of adipokines [59] and antioxidant capacity [50]. Third, uric acid levels increased significantly with obesity [60] and may have interfered in the FRAP assay [36]. Forth, the BMI of the mothers was not measured in this study and may also interfere by programming offspring for obesity [61]. The obesity in infants at birth also was not recorded. Fifth, it would be important to carry out analyzes stratifying groups below 12 months and above 12 months of age, however the sampe size was not suficient to demonstrated significant differences between the groups. Finally, a cross-sectional study was conducted, and it is important that further longitudinal studies be performed to clarify when other inflammatory changes are triggered.

## Conclusion

This is the first study that showed higher levels of leptin, adiponectin, BDNF, cortisol and lower levels of TBARS, SOD and CAT activities in overweight/obese infants when compared with their normal-weight peers. All these results point out neuroendocrine inflammatory response changes in overweight/obese infants between 6 and 24 months. Although there is already an environment that predisposes to a subsequent pro-inflammatory response, it is possible to demonstrate that neuroendocrine secretion changes occur to permit the control of the inflammatory process control in this age interval.
